# Maternal effects do not resolve the paradox of stasis in birth weight in a wild red deer populaton

**DOI:** 10.1111/evo.14622

**Published:** 2022-10-14

**Authors:** Julie Gauzere, Josephine M. Pemberton, Loeske E. B. Kruuk, Alison Morris, Sean Morris, Craig A. Walling

**Affiliations:** ^1^ Institute of Evolutionary Biology, School of Biological Sciences University of Edinburgh Edinburgh EH9 3FL UK; ^2^ Research School of Biology The Australian National University Canberra ACT 0200 Australia

**Keywords:** *Cervus elaphus*, evolutionary stasis, indirect genetic effects, natural selection, quantitative genetics, trade‐off

## Abstract

In natural populations, quantitative traits seldom show short‐term evolution at the rate predicted by evolutionary models. Resolving this “paradox of stasis” is a key goal in evolutionary biology, as it directly challenges our capacity to predict evolutionary change. One particularly promising hypothesis to explain the lack of evolutionary responses in a key offspring trait, body weight, is that positive selection on juveniles is counterbalanced by selection against maternal investment in offspring growth, given that reproduction is costly for the mothers. Here, we used data from one of the longest individual‐based studies of a wild mammal population to test this hypothesis. We first showed that despite positive directional selection on birth weight, and heritable variation for this trait, no genetic change has been observed for birth weight over the past 47 years in the study population. Contrarily to our expectation, we found no evidence of selection against maternal investment in birth weight—if anything, selection favors mothers that produce large calves. Accordingly, we show that genetic change in birth weight over the study period is actually lower than that predicted from models including selection on maternal performance; ultimately our analysis here only deepens rather than resolves the paradox of stasis.

One of the most puzzling and persistent problems in evolutionary ecology is the “paradox of stasis,” whereby heritable phenotypic traits under strong selection do not evolve (Merilä et al., 2001; Pujol et al., 2018). Many hypotheses have been formulated and investigated to explain the lack of evolutionary responses in wild populations, yet this paradox remains unresolved in most studies (except for a few notable examples, Merila et al. 2001; Kruuk et al. 2002; Reid et al. 2021). Some biological mechanisms have been identified as potentially interfering with evolutionary responses, such as the effect of population connectivity (Reid et al., 2021), indirect genetic effects (IGEs; Wolf et al. 1998), or multivariate constraints because of genetic correlations between traits (Teplitsky et al., 2014; Walling et al., 2014). Other explanations relate to statistical issues with parameter estimation (for breeding values, Postma 2006; or selection, Kruuk et al. 2002; Hadfield 2008). Although it is most likely that several mechanisms act simultaneously to generate or constrain responses the response to selection (Pujol et al., 2018), we still lack sufficiently detailed studies of specific biological mechanisms (e.g., IGEs) to evaluate their importance in driving evolutionary trajectories. Here, we focus on the evolutionary consequences of maternal effects ‐ potentially one form of IGE—for an important juvenile morphological trait, body weight. We provide a complete parameterization of a quantitative genetic model incorporating maternal effects (Cheverud, 1984) to predict the response to selection in a wild population.

Strong directional selection on phenotypic traits is commonly reported in natural populations (Kingsolver et al., 2001), especially on body size/mass, with selection typically favouring increased size (Kingsolver et al., 2012). This selection is often focussed on the juvenile stage, indicating that body size has greater fitness consequences for juveniles than adults (Rollinson & Rowe, 2015). However, despite ample heritable variation, microevolutionary stasis is often observed for body size (reviewed by Gotanda et al. 2015). One particularly promising hypothesis to explain stasis in body size is that positive selection on juveniles is counterbalanced by selection against parental investment in offspring growth, as formalized by the theory of “parent‐offspring conflict” (Lack, 1947). A few empirical studies have recently tested this hypothesis (Rollinson and Rowe, 2015; Thomson et al., 2017), but to date none has been able to predict the consequences of the parent–offspring conflict for the evolution of body size, and to compare it to the observed genetic change in body size, to explain microevolutionary stasis.

In many taxa, variation in maternal pre‐ and post‐natal investment is a major source of phenotypic variation in offspring traits (Mousseau & Fox, 1998), hereafter referred to as maternal effects. For convenience, maternal effects are often described as the overall effects of mothers on offspring phenotypes and are analyzed using a so‐called “variance partitioning” approach, which does not require the identification of the specific maternal traits underlying maternal performance. Maternal effects can only have evolutionary consequences for offspring traits if they are underlain by genetic differences between mothers (indirect genetic effects; Willham 1972; Kirkpatrick and Lande 1989). The genetic architecture of maternal effects itself has the potential to constrain the evolution of offspring traits, if maternal genetic effects covary with direct genetic effects ($\sigma_{AMg}$; Willham 1963, 1972; Wolf et al. 1998). In particular, in the absence of selection on maternal performance, a negative direct‐maternal genetic covariance can act as an evolutionary constraint. The genetic architecture of maternal effects has only been characterised in a few wild study systems (e.g., McAdam et al. 2002 in red squirrels, Wilson et al. 2005 in Soay sheep, Gauzere et al. 2020 in red deer). Thus, we lack sufficient empirical estimates to make general conclusions about its evolutionary consequences, although negative values of $\sigma_{AMg}$ have been reported in the animal breeding literature (Wilson & Reale, 2006). However, Cheverud (1984) was the first to highlight that natural selection likely operates on both offspring trait and maternal performance for the offspring trait. In such case, the evolutionary change in an offspring trait affected by maternal effects is:

$$\Delta z=\left(\sigma_{A}^{2}+\frac{1}{2}\sigma_{\mathrm{Mg}}^{2}+\frac{3}{2}\sigma_{\mathrm{AMg}}\right)\beta_{o}+\left(\sigma_{\mathrm{Mg}}^{2}+\sigma_{\mathrm{AMg}}\right)\frac{1}{2}\beta_{m}$$

where $\sigma_{A}^{2}$ is the additive genetic variance, $\sigma_{Mg}^{2}$ is the maternal genetic variance, $\sigma_{AMg}$ is the covariance between direct and maternal genetic effects, $\beta_{o}$ is the selection gradient on offspring trait, and $\beta_{m}$ is the selection gradient on maternal performance. Therefore, the evolution of traits with maternal effects can be seen as a special case of the joint evolution of correlated traits (Cheverud, 1984; Wolf & Brodie III, 1998), where constraints may also arise from antagonistic selection pressures on traits ($\beta_{o}$ and $\beta_{m}$ of opposite signs, for example favoring increased trait values in offspring but decreased maternal investment in that same trait), rather than from antagonistic genetic covariances ($\sigma_{AMg}$ < 0; Kruuk et al. 2008).

The first indication that selection on maternal performance may often be negative comes from theoretical and empirical studies on the trade‐off between offspring size and offspring number (i.e., parental breeding success) (Smith and Fretwell 1974; empirical evidence mostly found in multiparous species, but also in uniparous, see Rollinson and Rowe 2015). More generally, several studies have highlighted that reproduction comes with high costs for mothers (Goodman, 2006; Huber et al., 1999; Hamel et al., 2009). In red deer for instance, we know that gestation and lactation have important adverse consequences for immunity (Albery et al., 2020; Gauzere et al., 2021), and for mothers' subsequent breeding success and survival (Froy et al., 2016). Until recently, no statistical framework existed to measure selection on maternal performance ($\beta_{m}$), as defined by the single‐trait maternal performance model proposed by Cheverud (1984). Kirkpatrick & Lande (1989) proposed a more complete and general maternal effect model, often termed the “trait‐based” approach, but this approach requires quantifying selection on each of the individual traits underlying maternal effects for a focal offspring trait. Thomson et al. (2017) developed and tested a new method to estimate $\beta_{m}$ that uses a variance partitioning approach and relates total maternal effects on the focal trait to the mother's own fitness. This method has the advantage of being empirically tractable in wild populations, where measuring individual maternal traits is logistically challenging, and sufficiently accurate to understand the evolutionary dynamics of an offspring trait affected by maternal effects. Thomson et al. (2017) used their approach to show negative selection on parental performance for chick mass in a blue tit population, consistent with the idea that producing large offspring is costly for the parents. However, they did not have information about the genetic change in body mass and its genetic architecture (including indirect genetic effects) to demonstrate empirically that maternal effects can explain the observed evolutionary change (or stasis) in this offspring trait.

In the present study, we use a long‐term individual‐based study to predict the evolutionary response of birth weight in a wild red deer (*Cervus elaphus*) population on the Isle of Rum, Scotland. In previous analyses, we have shown that maternal effects explain 35% of the total phenotypic variance in birth weight, that maternal effects are mostly genetic in origin, and that the correlation between direct and indirect genetic effects for birth weight ($cor_{A,Mg}$ = –0.057 [–0.282; 0.314]) does not decrease the evolutionary potential of this trait (Gauzere et al., 2020). If genetic architecture is not a constraint, then the only other route by which maternal effects could constrain trait evolution is via the presence of antagonistic selection. Given previous evidence of positive selection on birth weight in the population (Bonnet et al., 2019; Kruuk et al., 1999), this would require selection on maternal performance to be strongly negative. The present study tests this hypothesis, using a unique data set for more than 2000 calves born from 600 females. To date, no study has tested for microevolutionary change or stasis in birth weight in the red deer study population. However, we know that birth weight varies between cohorts, driven by within‐mother plasticity in response to spring temperature (Froy et al., 2019). We therefore first test for phenotypic and genetic change in birth weight over the 47‐year study period. We then use state‐of‐art statistical methods to measure selection on birth weight and the method recently proposed by Thomson et al. (2017) to estimate selection on maternal performance. To do this, we analyze the association between individual birth weight and fitness (or its components) in both sexes, and the association between a mother's effect on her offspring's birth weight and her own fitness. This last model considers both among‐mother associations (e.g., do females who produce larger calves have higher overall fitness?) and within‐mother associations (does producing a large calf affect a female's fitness the subsequent year?). Finally, using a fully parameterized quantitative genetic model, we provide a detailed picture of the evolutionary consequences of maternal effects.

## Material and Methods

### STUDY POPULATION

All data were collected in the 12 km^2^ North Block of the Isle of Rum (Scotland; 57° 03′N, 06° 21′W) between 1972 and 2019. Deer living in this study area are recognizable either from ear tags or by facial characteristics, and individuals are closely monitored throughout the year. Mortality searching provides accurate information on death date for most resident individuals. There is no culling in the study area, but individuals are occasionally shot if they range elsewhere on the island. Most calves are caught soon after birth (often within 24 h) to be weighed, measured, tagged, and sampled for genetic analysis. Based on census data, each individual can be categorized as living in one of six regions of the study area (see Stopher et al. 2012). The population pedigree was reconstructed using genotype data for 440 single nucleotide polymorphisms and the R‐package SEQUOIA (see Huisman 2017). The maximum length of lineages in the pedigree is 11 generations, and it includes 4649 mother‐offspring links, with an average maternal sibship size of 4.7 calves.

In all analyses of the phenotypic traits described below, we restricted data sets to individuals with complete life‐histories, i.e., individuals with recorded birth and death years.

### PHENOTYPIC TRAITS

We only analysed individuals with complete life‐histories, i.e., individuals with recorded birth and death years. We considered the following variables:

***Birth weight***. Following previous analyses, we analyzed the weight of calves caught within 7 days of birth and born before August 1^st^ each year (Gauzere et al. 2020; 5 % of the data excluded). Models of birth weight used the capture weight of the calf as a response variable and accounted for the effects of the age at capture (in hours) as a covariate. An estimate of birth weight was also generated by correcting the measurement for age, based on the regression of weight on age (slope: 0.015 kg/h), to be included as a covariate.

***Survival***. We analyzed juvenile survival as a calf's chances of survival to the age of 2, that is, from birth to 1^st^ May, 2 years later, for the calves born up to 2017. Very few animals die between the ages of 2 and 3 unless they are shot, and after the age of 2, males may disperse from the study area. Information on these dispersers becomes less complete. For mature adults (i.e., individuals above the age of 3), annual survival was evaluated from the 1^st^ of May to the same date the following year. For individuals who were shot, data were recorded and analyzed up to the year prior to death.

***Adult annual breeding success (ABS)***. From observational and pedigree data, we calculated the number of offspring calved/sired each year by an individual from the age of three (maturity) to death. Females can give birth to a single calf per year, but males can mate multiply each rutting season. These data only recorded the ABS of males seen during the rut the year before.

***Lifetime breeding success (LBS)***. Lifetime breeding success was estimated for both sexes as the total number of calves produced across an individual lifetime. This data set includes individuals that died as juveniles and never get to reproduce. LBS was estimated for all the individuals born within the study area in the cohorts 1972–2005 that either died of natural causes or were still alive in 2019 (the latter category contained 7 individuals in total). Only the males seen in the study area during the rut most of their lifetime were included in this data.

### STATISTICAL ANALYSES

All models were fitted using a Bayesian framework, with the R‐package MCMCglmm v2.32 (Hadfield, 2010), which allowed us to propagate uncertainty in parameter estimates when measuring and predicting the response to selection. We considered our estimates significant if the 95 % credible intervals of their posterior distribution did not overlap zero. Table 1 provides the sample sizes analysed.

***Birth weight model***. We used a univariate animal model to decompose the genetic and environmental basis of birth weight. The fixed effects included the effect of calf sex, age at capture (in hours), maternal age (in years; linear and quadratic effects), and maternal breeding status in the previous year, i.e. whether she (i) calved and the calf survived to at least May 1 the year after birth (milk), (ii) calved and the calf died during the winter after birth, between October 1 and May 1 (winter yeld), (iii) calved and the calf died during the summer, before October 1 (summer yeld), (iv) female did not calve (true yeld), and (v) had never calved before (naive) (following Gauzere et al. 2020). We also accounted for birth location (a 6‐level factor; Stopher et al. 2012), and population density (the total number of females recorded in the year of birth). We considered four random effects, allowing us to decompose the phenotypic variance not explained by the fixed effects into direct additive genetic variance ($\sigma_{A}^{2}$), maternal genetic effect variance ($\sigma_{Mg}^{2}$), maternal environmental effect variance ($\sigma_{Me}^{2}$), and variance due to cohort effects ($\sigma_{C}^{2}$). Because birth weight was measured once per individual, permanent environmental effects were not fitted separately from residual effects. We also modeled the covariance between direct additive and maternal genetic effects ($\sigma_{\mathrm{AMg}}$), but we neglected the covariance with maternal environmental effects since these effects are negligible (Gauzere et al., 2020). The partitioning into genetic and non‐genetic factors relies on the relatedness information derived from the population pedigree (Kruuk, 2004). Body weight was treated as a Gaussian response variable. Such a 'variance partitioning' approach has been very useful for investigating maternal effects, as it does not require us to identify the actual maternal traits that explain maternal performance for birth weight, but it rather considers maternal effects as a general feature of the mother (Willham, 1972).

Further models using birth weight as a response variable to test for genetic change and to estimate selection used the same model structure as presented here.

***Testing for phenotypic and genetic change***. We fitted the birth weight model to measure the observed change in breeding values over the study period (1972‐2019). We included year as a fixed covariate in our model to avoid confounding phenotypic changes due to the plastic response to some environmental covariate with genetic change due to a response to selection (Postma, 2006). Using this model, we estimated the best linear unbiased predictors (BLUPs) for the calves' breeding values (a) and for the females' maternal genetic values (m_g_) for birth weight. Total genetic effects for birth weight, including maternal genetic effects, were estimated as: $a_{tot}=a+\frac{1}{2}m_{g}$. For each sample of the posterior distribution, we fitted linear regressions of the breeding values (*a*, $m_{g}$ and $a_{tot}$) against offspring birth year. We thus generated a posterior distribution for the linear slopes of genetic change, which allowed us to test for change over time while accounting for the uncertainty in BLUP estimation (Hadfield et al. 2010). We transformed this estimate of the annual genetic change for birth weight into one for genetic change per generation, considering a generation time of 8 years (following Bonnet et al. 2019), to give an estimate directly comparable to the predicted response to selection (from eq. (1) and (2)). To test for phenotypic change in birth weight, we used the raw data and simply tested a linear effect of the year of measurement on the observed data.

***Selection analyses***. In order to assess selection on birth weight and on maternal performance for birth weight, we investigated the connections between an individual's birth weight and its fitness, and between maternal performance for birth weight and the mother's own fitness. We considered two fitness proxies: (1) lifetime breeding success, and (2) annual fitness components, namely adult annual breeding success and annual survival. Analysis using annual components allows the inclusion of shot individuals, increasing our sample size, and also allow us to decompose selection via breeding success versus survival. In addition, annual fitness components can be modeled as binary traits (0/1; except for male ABS), whereas modeling LBS is notoriously challenging (de Villemereuil, 2018). However, the analysis of annual fitness components does not provide a straightforward estimate of selection gradients (β ; Thomson et al. 2017), while the analysis of (relative) LBS does (Lande, 1979).

**Figure 1:**
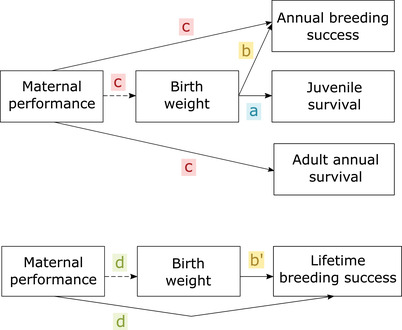
Diagram showing the associations between the traits and fitness components considered in the models (a)–(d). Solid lines represent the effect of an individual trait on its own fitness (e.g., maternal investment in birth weight affects a mother's own breeding success, or birth weight affects the chance of survival of a calf). Dashed lines represent the effects of mothers on their offspring (e.g., maternal effects affect offspring birth weight). The first panel shows the associations considering annual survival and annual breeding success as fitness proxies. The second panel shows the associations with lifetime breeding success. The colours letters show the four different models used in the analyses. Note that while model (b) investigates the effect of birth weight on an individual's own breeding success over its lifetime, model (c) investigates that consequences of maternal investment in birth weight for the mother's subsequent breeding success and survival (in the year t+1).

We used a series of univariate and bivariate mixed models (referred to as models a‐d; details below) to investigate the different phenotypic associations required for full representation of selection pressures. These associations are represented in Figure 1.

***Model (a): Effect of birth weight on juvenile survival***. Model (a) was a univariate model with juvenile survival as a response variable and birth weight as a covariate. The model also considered the effect of offspring sex, maternal age (linear and quadratic effects), spatial region, population density, offspring birth date, and maternal reproductive status as fixed effects potentially affecting juvenile survival. Birth year (or cohort) was included as a random effect to account for temporal heterogeneity in survival rates. A binomial model with a probit function (‘threshold’ model) was used to analyze this binary trait.

We also extended model (a) to allow the effect of birth weight on juvenile survival to vary between years, by defining an interaction term between birth weight and cohort (treated here as a fixed effect), thereby modeling the potential for fluctuating selection on birth weight.

***Model (b): Effect of birth weight on ABS and LBS***. Based on previous evidence, we assumed that late‐life fitness effects of birth weight could only occur through its effect on breeding success (as no effect has been reported on adult longevity; Kruuk et al. 1999). Thus, model (b) was a univariate model with ABS as a response variable and the individual's own birth weight as a covariate (Figure 1). We fitted one model for each sex, because of the different distributions of male and female ABS. Female ABS was treated as a threshold response variable, and male ABS as a Poisson response variable. For both sexes, we accounted for the effects of the individual's age on breeding success (linear and quadratic effects) and of the population density. For females only, we also included spatial region. We also considered the effect of the year of measurement of ABS and individual identity as random factors, because of the repeated measures on both years and individuals. We also tested the effects of birth weight on relative LBS, that is, individual LBS divided by the overall population mean. Relative LBS was treated as a Gaussian response variable (see Supporting Information Part S1 for more details).

***Model (c): Association between maternal investment and mother's breeding success and survival***. Model (c) was a bivariate model with response variables of the birth weight of the calf produced by a mother (in a given year *t*) and the mother's subsequent annual breeding success (ABS) or survival (SurvA) (in the year t+1; Figure 1). Birth weight was analysed using the same fixed and random effects as in the birth weight model described above, except that we did not split maternal effects into genetic and environmental components, but instead modelled total maternal effects as $m=m_{g}+m_{e}$ (using maternal identity as a random effect). The model for female annual breeding success/survival accounted for the effect of age (linear and quadratic effects), population density, and spatial location of the female during spring (year *t*; for ABS only), as well as random effects of the year of measurement and maternal identity as random effects. As offspring birth weight and mother's annual breeding success are repeated measurements at the level of the mother, this model thus defines a covariance structure between birth weight and fitness at both the among‐mother and within‐mother levels (i.e., covariances at the maternal effect level and residual level). These two covariances respectively capture the association between mothers' average maternal effect for birth weight and their average for the annual fitness component, and the association between the deviation in birth weight from the average maternal effect and the mother's average prospect of surviving or reproducing the following year. Female ABS and annual survival were treated as a threshold response.

***Model (d): Association between maternal investment and mother's LBS***. Finally, we fitted a bivariate model of birth weight of a mother's offspring and mother's (relative) LBS. The model used for birth weight was the same as model (c), and for female relative LBS it included the effect of cohort as a random effect. A female thus had one value of LBS and can have zero, one or several values for the birth weight of the offspring it calved. In model (d), the residual effects for LBS were allowed to covary with the maternal random effect for birth weight ($\sigma_{BW_{M},LBS_{R}}$) using the “covu = TRUE” model structure in MCMCglmm. $\sigma_{BW_{M},LBS_{R}}$ estimates the among‐female covariance between LBS and offspring birth weights and was then used to estimate selection on maternal performance for birth weight (see below).

A major assumption when measuring phenotypic selection is that the associations between traits and fitness are causal (Morrissey et al., 2010). We attempted to test this hypothesis by decomposing the phenotypic associations measured by models (a) to (d) into genetic and environmental components. These models are presented in Supporting Information Part S2.

**Table 1:** Description and summary statistics for the models and traits analysed in this study. Capture weight is the weight of calves caught within 7 days of birth. Birth weight was estimated based on the regression of weight on age (slope = 0.015 kg/h) and used as a covariate in models (a) and (b). Binary traits take specific values, reported in “{ }.”

	Units	n	Mean	Range	CV
*Model of genetic change*					
Capture weight	kg	2484	6.91	[1.9; 13.2]	0.20
*Model (a)*					
Birth weight	kg	2302	6.41	[1.9; 11.0]	0.20
Juvenile survival	rate	2302	0.50	{0;1}	1.00
*Model (b)*					
Birth weight	kg	2302	6.41	[1.9; 11.0]	0.20
Annual breeding success ♀	calves	574	0.60	{0;1}	0.82
Annual breeding success ♂	calves	349	0.75	[0; 14]	2.03
*Model (c)*					
Capture weight	kg	2444	6.91	[1.9; 13.2]	0.20
Annual breeding success ♀	calves	605	0.56	{0;1}	0.88
Annual survival ♀	rate	638	0.90	{0;1}	0.33
*Model (d)*					
Capture weight	kg	1724	6.91	[1.9; 13.2]	0.20
Lifetime breeding success ♀	calves	907	2.64	[0; 14]	1.39

### PREDICTING THE RESPONSE TO SELECTION

Because the approaches using LBS and annualized fitness components provide qualitatively similar results (see Results section), we only used the outputs of the models using relative LBS as a response variable to predict the response to selection. We estimated directional selection on birth weight as the linear regression coefficient of relative LBS on birth weight ($\beta_{o}$) for both sexes, using the output of model (b). We estimated the selection gradient on maternal performance for birth weight as: $\beta_{m}$ = $\sigma_{BW_{M},LBS_{R}}/\sigma_{m}^{2}\left(BW\right)$, with $\sigma_{m}^{2}\left(BW\right)$ being the maternal effects variance for birth weight, using the output of model (d). Variance‐standardized selection gradients ($\beta_{SD}$) were also estimated, with respect to the phenotypic standard deviation of birth weight, to provide a dimensionless measure of selection that allows comparison across traits and studies.

We first predicted the response to selection of birth weight, neglecting the evolutionary consequences of maternal effects, by using the univariate version of Lande's equation (Lande, 1979):

(1) $$\Delta \bar{z}=\sigma_{A}^{2}.\beta_{o}$$

where $\Delta \bar{z}$ is the predicted genetic change after one generation and $\sigma_{A}^{2}$ the direct additive genetic variance for birth weight and $\beta_{o}$ is the direct selection gradient.

Then, we predicted the response to selection accounting for maternal effects on birth weight (Cheverud, 1984), as:

(2) $$\Delta \bar{z}=\left(\sigma_{A}^{2}+\frac{1}{2}\sigma_{\mathrm{Mg}}^{2}+\frac{3}{2}\sigma_{\mathrm{AMg}}\right)\beta_{o}+\left(\sigma_{\mathrm{Mg}}^{2}+\sigma_{\mathrm{AMg}}\right)\frac{1}{2}\beta_{m}$$

where $\sigma_{Mg}^{2}$ is the maternal genetic variance for birth weight and $\sigma_{\mathrm{AMg}}$ the covariance between direct and maternal genetic effects and $\beta_{m}$ is the selection gradient on maternal performance. All the (co)variance components used in eq. (1) and (2) were estimated from the birth weight model presented above.

Model 2 accounts for the fact that antagonistic genetic covariance ($\sigma_{\mathrm{AMg}}$ <0) and antagonistic selection ($\beta_{o}$ and $\beta_{m}$ of opposite signs) can impose constraints on the evolutionary response of birth weight. Maternal effects interfere with evolutionary responses if |$\Delta {\bar{z}}_{eq.2}$| <|$\Delta {\bar{z}}_{eq.1}$|. With $\sigma_{\mathrm{AMg}}∼$ 0 (Gauzere et al., 2020) and $\beta_{o}$ likely being positive (Kruuk et al., 1999), maternal effects can only explain evolutionary stasis for birth weight if $\beta_{m}$ is strongly negative.

## Results

### OBSERVED PHENOTYPIC AND GENETIC CHANGE

**Figure 2:**
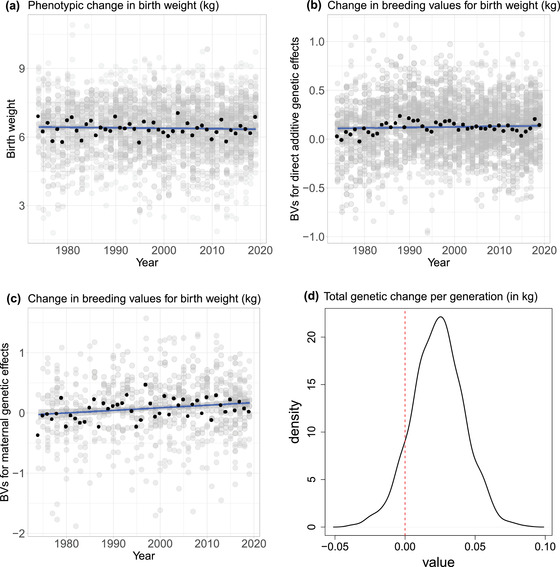
Observed phenotypic (panel a) and genetic change (b, c) for birth weight (in kg) over the study period, from 1972 to 2019. Change in breeding values (BVs) for direct genetic effects (b) and maternal genetic effects (c) are represented separately. Breeding values for maternal genetic effects are plotted against the mean offspring birth year. (d) Posterior distribution for the predicted total genetic change in birth weight (in kg), accounting for both direct and maternal genetic effects (equation 2).

We found no significant phenotypic change for birth weight over the study period (slope = −0.002 kg/year, p‐value = 0.32; Figure 2a). This phenotypic stasis could be concealing genetic changes if a plastic response of birth weight exactly counteracts the response to selection. Using a Bayesian animal model to extract breeding values for birth weight, we found no evidence of genetic change over time: the estimated slope of linear change in breeding values across years was 0.0008 [−0.0028 to 0.0046] kg/year for direct genetic effects and 0.0029 [−0.0020 to 0.0099] kg/year for maternal genetic effects (values in brackets provide the 95 % credible intervals; Figure 2, Table S1). Over the 47 years analysed, the total resulting change in breeding values was $g_{tot}$ = 0.148 [−0.054 to 0.356] kg, that is a 2.3 % change in birth weight mean.

### GENETIC ARCHITECTURE OF BIRTH WEIGHT

**Figure 3:**
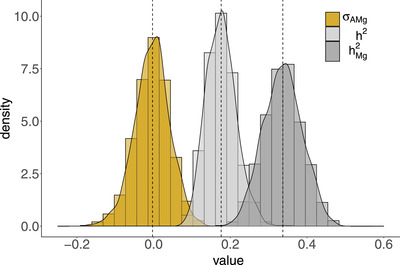
Posterior distributions for the proportion of variation in birth weight explained by genetic differences among calves (i.e., direct additive genetic effects), estimated as $h^{2}=\sigma_{A}^{2}/\sigma_{P}^{2}$ (light grey), and variance explained by maternal genetic effects, as $h_{Mg}^{2}=\sigma_{Mg}^{2}/\sigma_{P}^{2}$ (dark grey), with $\sigma_{P}^{2}$ the total phenotypic variance in birth weight. We also represent the estimated covariance between direct and maternal genetic effects ($\sigma_{\mathrm{AMg}}$; yellow). The dotted lines represent the median value of each distribution.

We also estimated the proportion of variation in birth weight explained by the random effects included in the model (Figure 3). Consistent with Gauzere et al. (2020), we found that maternal genetic effect variance was the main source of phenotypic variance (after correcting for fixed effects), and maternal environmental effects only explained 0.2 [0 to 11] % of the phenotypic variance. The additive genetic effect variance between calves explained 18 [11 to 26] % of the phenotypic variance in birth weight, and the estimated covariance between additive and maternal genetic effects was very small and centred close to zero ($\sigma_{\mathrm{AMg}}$ = 0.015 [−0.090 to 0.091]; Figure 3).

### DIRECTIONAL SELECTION ON BIRTH WEIGHT

**Figure 4:**
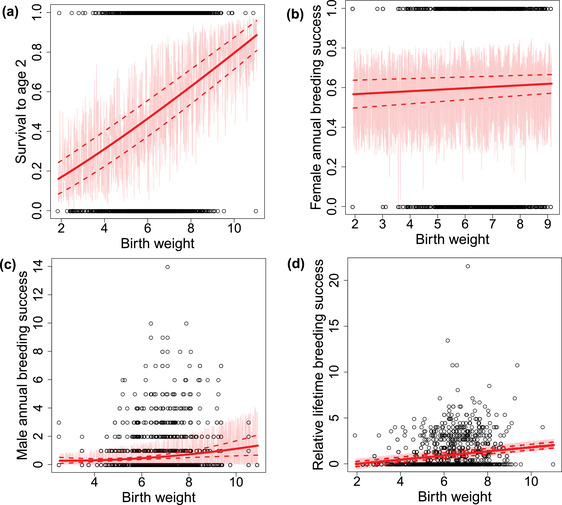
Effect of birth weight on juvenile survival (panel a) and on adult annual breeding success in males and females (analyses run separately between sexes; panels b and c), as estimated from models (a) and (b). We also represent the effect of birth on relative lifetime breeding success (including both sexes; panel d). The black circles represent the observed data and the light red lines represent the values of fitness for a given birth weight predicted by our Bayesian models. The solid red line represents the fitted non‐linear curve into these predictions, and the dotted lines the 95 % credible interval around the predictions.

**Figure 5:**
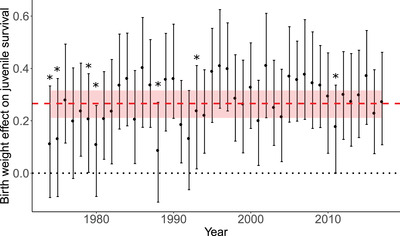
Temporal variation in the effect of birth weight on juvenile survival. The red dashed line represents the average effect of birth weight on juvenile survival (estimated from model (a)). The black stars indicate the years for which the slope was significantly different from this mean effect (estimated using a model that first fitted an effect of birth weight and its interaction with years).

As expected, from model (a), we found a positive linear effect of birth weight on juvenile survival (slope: 0.266 [0.211 to 0.316]; Figure 4a), meaning that heavier calves had a higher probability of reaching the age of 2. This effect of birth weight on juvenile survival was relatively constant through time (only seven years for which this effect departed from the mean; *p‐value* < 0.05) and we found no fluctuation of the sign of selection on birth weight (Figure 5).

Outputs of model (b) showed no evidence of an effect of birth weight on female ABS (slope: 0.017 [−0.021 to 0.056] ; Figure 4b). However, we found a positive linear effect of birth weight on male ABS, with males that were heavier as calves having higher ABS as adults (slope: 0.195 [0.062 to 0.325]; Figure 4c). Consistent with the effect of birth weight on juvenile survival and ABS, we found a positive effect of birth weight on relative LBS (combining both sexes), with an estimated linear selection gradient on birth weight of $\beta_{o}$ = 0.30 [0.22 to 0.39] (Figure 4d, Table S3). The variance‐standardized selection gradient, $\beta_{oSD}$ = 0.34 [0.24 to 0.45], is larger than previous meta‐analytic estimates of the strength of selection on juvenile size across multiple systems (median $\beta_{SD}$ = 0.22 reported by Rollinson and Rowe 2015). See the tables in SI for the fixed effects of these models.

### DIRECTIONAL SELECTION ON MATERNAL PERFORMANCE FOR BIRTH WEIGHT

**Figure 6:**
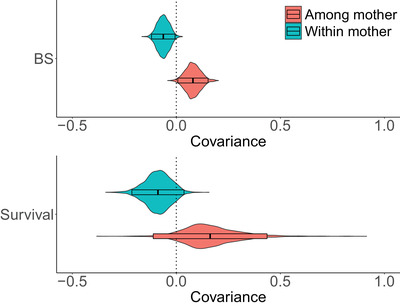
Posterior distributions of the phenotypic covariances between birth weight and adult annual breeding success/survival, as estimated in model (c), with boxes representing the median and 95 % credible intervals. This covariance structure was defined at the among mother level and within‐mother level. The covariances between adult annual breeding success (BS) and birth weight are different from zero (boxes do not overlap zero). The covariances between adult annual survival and birth weight are estimated with a larger uncertainty, with plausible values including both positive and negative trends.

From model (c), we investigated the costs/benefits of maternal investment in birth weight, decomposing its potential effects on mothers' breeding success and survival. There was a positive association between offspring birth weight and maternal ABS${}_{\left(t+1\right)}$ at the among‐mother level, with $\sigma_{M}\left(BW,ABS_{\left(t+1\right)}\right)$ = 0.067 [0.010 to 0.152], meaning that females who consistently produced large calves also had the largest number of calves. We also found a negative association between offspring birth weight and maternal ABS at the within‐mother level, with $\sigma_{R}\left(BW,ABS_{\left(t+1\right)}\right)$ = −0.079 [−0.118 to −0.010] (Figure 6), meaning that increased maternal investment in birth weight in a given year reduced females' chances of reproduction the following year. We found a similar pattern for the association between offspring birth weight and maternal survival, but the estimated covariance terms were never different from zero, with $\sigma_{M}\left(BW,SurvA_{\left(t+1\right)}\right)$ = 0.111 [−0.080; 0.459] and $\sigma_{R}\left(BW,SurvA_{\left(t+1\right)}\right)$ = −0.071 [−0.215 to 0.025]. The larger uncertainty in parameter estimates is consistent with the fact that survival data is less variable than breeding success (Table 1).

We then investigated the consequences of maternal performance for mother's (relative) LBS using model (d). The estimated covariance between maternal effects for birth weight and residual effects for relative LBS was positive, marginally overlapping zero, with $\sigma_{BW_{M},LBS_{R}}$ = 0.15 [−0.02 to 0.27]. The resulting selection gradient on maternal performance was $\beta_{m}$ = 0.28 [−0.05 to 0.51]. The variance standardized selection gradient, $\beta_{mSD}$ = 0.28 [−0.04 to 0.58], is in the upper range of the values reported in the literature (Kingsolver et al., 2001).

### PREDICTED EVOLUTIONARY RESPONSE

**Figure 7:**
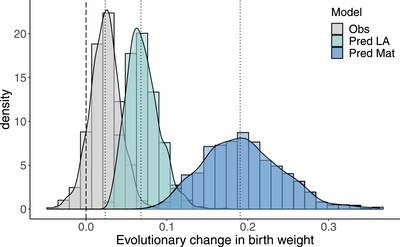
Posterior distributions of the observed and predicted evolutionary responses for birth weight per generation. The dotted lines represent the posterior mode of the distributions. “Pred LA” represents the response to selection predicted by the Lande's equation (see Eq. 1), while “Pred Mat” represents the response predicted using a model that also accounts for the evolutionary consequences of maternal effects (Eq. 2). The large uncertainty “Pred Mat” is explained by the high estimated error in $\beta_{m}$. Considering that the average birth weight of a calf is 6.4 kg, we therefore observed 0.4 % change in the mean per generation, while we predicted 1% change in the mean per generation using Eq. 1, and 3% change per generation using Eq. 2.

Finally, we compared the predicted and observed responses to selection for birth weight per generation. Using a model that neglected the evolutionary consequences of maternal effects (eq. 1), we predicted a positive genetic change with a point estimate more than 2 times higher than the observed one (0.064 [0.031 to 0.105] instead of 0.026 [−0.010 to 0.063] kg per generation; Figure 7). When we accounted for maternal effects (using eq. 2), we found an even higher discrepancy between the observed and predicted evolutionary responses, with a predicted response of 0.195 [0.100 to 0.278] kg per generation (Figure 7), which is very fast evolution (3 % change in the mean per generation). Accounting for selection on maternal performance also increased the uncertainty in the predicted response to selection because of the large uncertainty in the estimation of $\beta_{m}$.

## Discussion

Despite positive directional selection on birth weight, and heritable variation for this trait, we observed no genetic change for birth weight over the past 47 years in the Isle of Rum red deer population. One possible explanation for this evolutionary stasis is the hypothesis of “parent‐offspring conflict” (Rollinson and Rowe, 2015). Although promising, the hypothesis has never been fully tested in a wild population, possibly because it requires both an understanding of the genetic architecture of parental effects and estimates of selection on parental performance (Cheverud, 1984; Kirkpatrick & Lande, 1989), information that is usually only accessible for experimental studies (cross‐fostering; Hadfield et al. 2013; Thomson et al. 2017) and long‐term individual‐based studies (Wilson et al. 2005; this study). Based on our understanding of the genetic basis of maternal effects for birth weight in our red deer study population (Gauzere et al., 2020), we predicted microevolutionary stasis may occur because of strong negative selection on maternal performance. However, we found no evidence of antagonistic selection on birth weight and maternal performance for this trait, with selection on maternal performance being positive and as strong as direct selection on birth weight, although estimated with greater uncertainty. To our knowledge, this study represents the first attempt to combine estimates of selection on the offspring trait, and maternal performance for the trait, with estimates of (co)variation between direct and maternal genetic effects to demonstrate empirically that maternal effects do not constitute an evolutionary constraint.

A recent meta‐analysis has provided support for the offspring size/number trade‐off in a variety of taxa, including mammals (Rollinson and Rowe, 2015). In uniparous species, such as red deer, such a trade‐off can only exist between current and future reproductive effort/success (Williams, 1966). Thanks to the close monitoring of individuals throughout their life, we were able to decompose the association between phenotypic traits and fitness at both the within‐ and among‐mother levels using a multivariate approach. We found that mothers producing large calves pay a cost in terms of their own breeding success the following year (association measured at the within mother level), consistent with life‐history theory (Lack, 1947) and with previous results on the study population (Clutton‐Brock et al., 1989). However, we found that mothers that on average produce heavier calves also produce more calves. This positive association measured at the among‐female level can be interpreted as selection on maternal performance. This is supported by the analysis of lifetime breeding success, showing a positive covariance between maternal performance and female lifetime breeding success. Combined these results suggest that selection on mothers is acting to increase maternal investment in birth weight.

A key assumption when measuring phenotypic selection is that a causal relationship exists between trait and fitness (Kruuk et al., 2003; Morrissey et al., 2010). Although we expect maternal investment in birth weight to have direct fitness consequences, we cannot test for causation using our correlative approach. Consequently, the relationships between traits and fitness might also be due to non‐causal environmental factors affecting the traits and fitness (Morrissey et al., 2010). This might explain both why there is no response to the apparent selection on birth weight and why no cost is visible when analysing permanent differences in mothers' life‐history traits (as often observed in the literature; Wilson and Nussey 2010). However, for our measures of selection on maternal performance, unmeasured environmental covariance seems unlikely given that we estimated that the vast majority of variance in maternal effects was genetic in origin and that the environmental variance in maternal effects was small (see also Gauzere et al. 2020). When we attempted to dissect phenotypic associations into genetic and environmental components, the model fails due to a lack of environmental maternal effect variance and thus covariance with fitness (see SI Part S2 for more information). Nonetheless, variation in individual resource acquisition (often termed individual condition or quality) might have a genetic basis and thus mask any potential trade‐off between maternal investment for birth weight and the mother's own fitness (van Noordwijk & de Jong, 1986). For this reason, our approach may underestimate the cost of maternal investment (Thomson et al., 2017), but critically this cannot explain the lack of evolutionary response as genetic basis of among‐mother variation in performance for birth weight would still facilitate an evolutionary response even if its origin was genetic variation in individual quality or condition.

A number of solutions have been proposed to circumvent the issue of variation in individual quality masking apparent costs of parental care, including: (1) directly measuring the quality of individuals and accounting for it in models (Lim et al., 2014) or (2) using experimental manipulation of parental care to effectively measure the cost of caring (e.g., by altering brood/clutch size; Santos and Nakagawa 2012). Realistically, neither of these options could be implemented in our study system. However, our models included the effect of the spatial region to account for the variation in habitat quality in the study area, which potentially underlie part of the between‐individual variation in quality. Moreover, the only study that has estimated negative selection on parental performance has found a cost of postnatal investment for parents' survival (in blue tits; Thomson et al. 2017). Our study focuses on the fitness consequences of prenatal maternal investment, and not postnatal investment, which might explain the different results. In red deer, we know that mothers invest a lot in care after birth, and especially in mammals, we know that lactation is often more costly for females than gestation (Clutton‐Brock et al., 1989; Froy et al., 2016). Therefore, it is possible that pre‐natal investment might not be costly enough to constrain the evolution of birth weight.

Thomson et al. (2017) showed that selection on maternal performance can be accurately estimated using a variance partitioning approach, as used here, if selection on the maternal traits underlying maternal effects is proportional to their maternal effect. This approach makes other assumptions, like the fact that selection is constant across generations. Indeed, maternal effects are modeled as the overall effects of mothers on offspring phenotypes, integrated over a female's lifetime. Consequently, it is not possible to model fluctuating selection on maternal performance with such an approach. Doing so would require a “trait‐based” approach and to measure the maternal traits underlying maternal effects (Kirkpatrick and Lande, 1989; McGlothlin & Galloway, 2014), information that is not always easily accessible in wild study systems (but see a successful application by McAdam and Boutin 2004). Nonetheless, we tested the validity of this hypothesis for the direct component of selection and found no evidence of temporal fluctuation in either the sign or intensity of selection on birth weight. Most importantly, McGlothlin and Galloway (2014) showed that the differences in the predictions of “variance partitioning” vs “trait‐based” models were quite subtle, especially in the absence of cascading maternal effects, that is, when maternal effects are not mediated by traits that are themselves maternally influenced (Pick et al., 2019). Neglecting fluctuating selection and cascading maternal effects might affect our predictions, but without the constraint imposed by the genetic architecture or maternal selection ($\sigma_{AMg}$ < 0 or $\beta_{m}$ < 0), they are unlikely to resolve the paradox of stasis on their own (McGlothlin and Galloway, 2014).

Using a fully parameterized model that includes maternal effects leads to a stronger discrepancy between the predicted and observed genetic change for birth weight than using a simple model of evolutionary change. This is in large part because maternal effects provide additional heritable genetic variation on which selection can act (see Gauzere et al. 2020), as also reported in other species (McAdam et al., 2002; McAdam & Boutin, 2004; Pick et al., 2016; Wilson & Reale, 2006). Therefore, maternal effects do not appear to resolve the paradox of stasis observed in our study system. The question remains, what biological mechanism could explain stasis? A commonly advanced hypothesis, that cannot be validated or rejected here, is that genetic correlations between the focal trait and other traits under selection constrain the evolutionary response of birth weight (Morrissey et al., 2010; Teplitsky et al., 2014). However, we would not expect to see the genetic correlation between birth weight and fitness if this was true (Part S2 in Supporting Information). A more recent consideration is that social competition may be important for understanding the evolutionary trajectories of traits conferring competitive ability (Fokkema et al., 2021; Wolf et al., 1999; Wilson, 2014). Social competition has already been implicated in the evolutionary constraint of dominance in the study population (Wilson et al., 2011), but it might also be relevant to consider for traits like body mass/size (Formica et al., 2011). Competition can create positive feedback loops that accentuate environmental coupling of traits and fitness (Fokkema et al., 2021; Wilson, 2014). As with maternal genetic effects, genetic differences in the capacity of individuals to compete for resources generate indirect genetic effects, which can interfere with evolutionary responses (Fisher & McAdam, 2019; Wilson, 2014). Therefore, the results we find here—positive association between maternal performance and fitness, but evolutionary stasis for birth weight—are consistent with a competition‐based model of evolutionary constraint (Wilson, 2014). Testing this hypothesis is out of the scope of this study and would require characterizing social interactions or individuals' competitive ability, but it offers promising prospects to better understand the evolutionary trajectory of birth weight, and other resource‐dependent traits.

## Conclusions

This study is, to our knowledge, the first to fully parameterize a maternal effects model accounting for selection on maternal performance to explain the evolutionary dynamics of an offspring trait in a wild population. It is also one of the few to estimate selection on maternal performance, which requires statistical models that have only recently been suggested (Thomson et al. 2017). Contrary to theoretical and empirical expectations, we find no evidence of selection against maternal investment in birth weight. Maternal effects therefore do not appear to resolve the paradox of evolutionary stasis observed in this offspring trait. Our study shows that incorporating relevant biological mechanisms into quantitative genetic models can strengthen, rather than explain, the discrepancy between observed and predicted evolutionary responses.

## DATA ACCESSIBILITY STATEMENT

The data and code used in this paper have been deposited on Figshare, 10.6084/m9.figshare.20332407.

## AUTHOR CONTRIBUTIONS

J.G., J.M.P., and C.A.W. designed the study. S.M. and A.M. collected the field data. J.G. ran the analyses and drafted the manuscript. J.G., J.M.P., L.E.B.K., and C.A.W. discussed and interpreted the findings. J.G., J.M.P., L.E.B.K., and C.A.W. jointly contributed to revisions of the manuscript.

Associate Editor: A. McAdam

Handling Editor: T. Chapman

## Supporting information

Table S1: Linear mixed model results for each predictor tested in the model of genetic change for birth weight.
Table S2: Linear mixed model results for each predictor tested in model (a) to (d).
