# Supplementary material for: Maternal effects do not resolve the paradox of stasis in birth weight in a wild red deer populaton
Source: Evolution. 2022 Oct 14;76(11):2605–17. doi: 10.1111/evo.14622 (PMC9828841; doi:10.1111/evo.14622)
Supplement: Supplementary file 1 — Table S1: Linear mixed model results for each predictor tested in the model of genetic change for birth weight. Table S2: Linear mixed model results for each predictor tested in model (a) to (d). [file EVO-76-2605-s001.pdf]

Supporting Information for

Maternal effects do not resolve the paradox of stasis in birth weight in a wild mammal

Contents

1: Model of lifetime breeding success

2: Genetic and environmental covariance between birth weight, maternal performance, and fitness

3: Linear mixed model results for each predictor tested in the model of birth weight, and models (a)-(d)

Table S1: Model of genetic change for birth weight.

Table S2: Linear mixed model results for each predictor tested in model (a) to (d).

1: Model of lifetime breeding success

One of the most challenging aspects in modelling fitness, and particularly lifetime breeding success, is that it typically follows a non-Gaussian distribution. Some authors have therefore suggested that fitness should be analysed using more complex “generalised” linear models (de Villemereuil 2018, Bonnet et al. 2019). However, these models also rely on assumptions (e.g., strict mean-variance scaling relationship implied by the Poisson distribution), and violation of these distributional assumptions have unclear consequences for parameter estimates. On the contrary, we know that Gaussian models are robust to violations of distributional assumptions (Schielzeth et al. 2020), suggesting they may be more reliable to use than generalised linear models. As a result, we decided to analyse (relative) lifetime breeding success as a Gaussian response in models (b) and (d).

We also fitted a model for relative lifetime breeding success with sex-specific birth weight effects. However, birth weight affected the lifetime breeding success of females and males similarly (female slope : 0.22 [0.11; 0.36]; male slope: 0.25 [0.14; 0.37]). Therefore, for the sake of simplicity, we use in the main text a model including both sexes to estimate the linear selection gradient on birth weight (model (b)).

Finally, to predict the response to selection, we used the outputs of the models using relative lifetime breeding success, rather than annualised fitness components, as these provided a more straightforward estimate of the selection gradient (β).

2: Genetic and environmental covariance between birth weight, maternal performance, and fitness

A major assumption when measuring phenotypic selection is that the association between traits and fitness is causal. In particular, any environmental covariate that affects both a focal trait and fitness can lead to erroneously predict some evolutionary change in response to selection but cannot contribute to evolutionary change. Here, we investigated how such environmental covariance might explain the discrepancy between the observed and predicted response to selection presented in the main text. For this, we decomposed the phenotypic associations measured by models (a) to (d) into genetic and environmental components using bivariate models and relatedness information derived from the population pedigree.

First, we used a bivariate model between offspring birth weight and its juvenile survival to estimate the genetic and residual covariance between these two traits (thereby extending model (a)). We found positive covariances at both the genetic (cov_G(BW, JuvSurv) = 0.115 [0.02; 0.245]) and residual level (cov_R(BW, JuvSurv) = 0.20 [0.10; 0.28]). We did the same using a bivariate model with birth weight and lifetime breeding success as response variables (extending model (b)). We found a small positive genetic covariance between these two traits (cov_G(BW, LBS) = 0.044 [-0.10; 0.24]), with credible intervals widely overlapping zero. This result was consistent with the (absence of) observed change in breeding values for birth weight (Fig. 2). Indeed, according to the second theorem of selection, the evolutionary change should be equal to the genetic covariance of a trait with fitness (Robertson-Price identity; Roberston 1966 Anim. Prod. 8: 95:108, Price 1970 Evolution 45:853-861). These models included both sexes and accounted for the effect of sex as a fixed effect.

We then ran similar bivariate models to decompose the genetic and environmental associations between maternal performance for birth weight and a female's fitness. We relied on the same variance partitioning approach as presented in the main text to define maternal effects for birth weight and we split maternal effects into genetic and environmental components. First, we used the mothers' annual breeding success as a proxy of fitness and we modelled maternal genetic and maternal environmental effects for birth weight (extending model (c)). We found a positive genetic covariance between maternal effects for birth weight and mothers' breeding success (covG(MatEff, ABS) = 0.045 [-0.047; 0.15]) and a small positive covariation at the maternal environmental level (covE(MatEff, ABS) = 0.0003 [-0.027; 0.08]), indicating that the phenotypic association between these two traits is mostly driven by genetic effects. This result makes sense because almost all the maternal effect variance captured by our variance partitioning approach is due to genetic effects (Gauzere et al. 2020 10.1111/evo.14000). Second, we used lifetime breeding success as a proxy of fitness. We used a model structure very similar to model (d) but this time we defined covariance terms at the maternal genetic and environmental levels. This model showed poor convergence, with the MCMC chains not mixing well, suggesting that we lacked sufficient information in our data to properly fit such a complex model structure. The fact that the maternal environmental variance is almost null might also explain the poor fit of this model. This poorly fitted model nonetheless suggested a small positive genetic covariance between maternal effects for birth weight and mothers' LBS ($cov_G(BW_{Mat}$, LBS) = 0.25 [-0.17; 0.65].

Overall, these results suggest that the phenotypic association between traits, maternal effects and fitness are not solely driven by environmental effects, which means that an evolutionary response to selection should be expected (but is not observed).

3: Linear mixed model results for each predictor tested in the model of birth weight, and models (a)-(d)

Table S1: Linear mixed model results for each predictor tested in the model of genetic change for birth weight. 'Region' defines the birth location (following Stopher et al. 2012, Gauzere et al. 2020) and is relative to the value for 'IA' (Intermediate Area). 'Repro' refers to the reproductive status of the female the year before and is relative to the value for 'WinterYeld'.

	post.mean	l-95% CI	u-95% CI	pMCMC
Intercept	6.095	-17.670	26.360	0.5675
Calf sex [male]	0.315	0.242	0.382	<0.001 ***
AgeHrs	0.016	0.015	0.017	<0.001 ***
PopDens	-0.004	-0.008	-0.001	0.0187 *
Maternal age	0.458	0.391	0.535	<0.001 ***
Maternal age^2	-0.025	-0.028	-0.021	<0.001 ***
Repro [Milk]	0.209	0.085	0.353	0.0085 **
Repro [Naïve]	0.229	0.073	0.408	0.0051 **
Repro [SummerYeld]	0.698	0.546	0.850	<0.001 ***
Repro [TrueYeld]	0.637	0.503	0.774	<0.001 ***
Region [LA]	0.231	-0.052	0.553	0.1376
Region [MG]	0.703	0.475	0.968	<0.001 ***
Region [NG]	0.280	0.068	0.505	0.0153 *
Region [SG]	0.971	0.697	1.271	<0.001 ***
Region [SI]	0.119	-0.123	0.328	0.311
Year	-0.001	-0.011	0.011	0.8751

Table S2: Linear mixed model results for each predictor tested in model (a) to (d).

Model (a) of juvenile survival

	post.mean	l-95% CI	u-95% CI	pMCMC
Intercept	1.080	-0.253	2.617	0.142
Calf sex [male]	-0.366	-0.479	-0.244	0.001 ***
Birth weight	0.266	0.211	0.316	0.001 ***
Maternal age	0.125	0.002	0.259	0.054 .
Maternal age^2	-0.008	-0.014	-0.001	0.01 *
Region [LA]	0.732	0.484	0.951	0.001 ***
Region [MG]	0.885	0.677	1.078	0.001 ***
Region [NG]	0.207	0.028	0.377	0.032 *
Region [SG]	0.896	0.654	1.112	0.001 ***
Region [SI]	0.158	-0.024	0.346	0.092 .
Birth date	-0.018	-0.023	-0.012	0.001 ***
Repro [Milk]	0.331	0.099	0.541	0.004 **
Repro [Naïve]	0.256	-0.040	0.529	0.076 .
Repro [SummerYeld]	0.073	-0.180	0.332	0.554
Repro [TrueYeld]	0.225	0.022	0.455	0.038 .
PopDens	-0.004	-0.008	0.001	0.104

Model (b) of male annual breeding success

	post.mean	l-95% CI	u-95% CI	pMCMC
Intercept	-12.095	-13.946	-10.307	<0.001 ***
Age	1.969	1.702	2.202	<0.001 ***
Age^2	-0.096	-0.110	-0.083	<0.001 ***
Birth weight	0.195	0.062	0.325	0.002 **
PopDens	0.002	-0.003	0.008	0.392

Model (b) of female annual breeding success

	post.mean	l-95% CI	u-95% CI	pMCMC
Intercept	-0.790	-1.474	-0.093	0.028 *
Age	0.436	0.389	0.486	<0.001 ***
Age^2	-0.022	-0.025	-0.019	<0.001 ***
Birth weight	0.017	-0.021	0.056	0.384
Region [LA]	0.110	-0.063	0.302	0.242
Region [MG]	-0.034	-0.206	0.117	0.678
Region [NG]	-0.215	-0.347	-0.045	0.002 **
Region [SG]	0.100	-0.061	0.251	0.216
Region [SI]	-0.171	-0.327	-0.015	0.038 *
PopDens	-0.003	-0.006	-0.001	0.028 *

Model (b) of relative lifetime breeding success (both sexes together)

	post.mean	l-95% CI	u-95% CI	pMCMC
Intercept	0.072	-0.518	0.635	0.794
Calf sex [male]	-1.086	-1.275	-0.879	<0.001 ***
Birth weight	0.237	0.148	0.322	<0.001 ***

Bivariate model (c) of birth weight and female annual breeding success

	post.mean	l-95% CI	u-95% CI	pMCMC
CaptWt:Intercept	4.560	3.728	5.273	<0.001 ***
FecA:Intercept	0.153	-0.826	1.173	0.802
CaptWt:Calf sex [male]	-0.329	-0.400	-0.258	<0.001 ***
CaptWt:AgeHrs	0.016	0.015	0.017	<0.001 ***
CaptWt:Maternal age	0.450	0.381	0.526	<0.001 ***
CaptWt:Maternal age^2	-0.024	-0.028	-0.021	<0.001 ***
CaptWt:Repro [Naïve]	0.037	-0.116	0.199	0.6406
CaptWt:Repro [SummerYeld]	0.512	0.381	0.632	<0.001 ***
CaptWt:Repro [TrueYeld]	0.449	0.336	0.554	<0.001 ***
CaptWt:Repro [Winter Yeld]	-0.193	-0.333	-0.056	0.0102 *
CaptWt:Region [LA]	0.144	-0.135	0.407	0.2889
CaptWt:Region [MG]	0.629	0.415	0.871	<0.001 ***
CaptWt:Region [NG]	0.137	-0.057	0.362	0.2022
CaptWt:Region [SG]	0.857	0.618	1.099	<0.001 ***
CaptWt:Region [SI]	0.044	-0.156	0.269	0.6746
CaptWt:PopDens	-0.002	-0.005	0.001	0.2379
FecA:Maternal age	0.323	0.230	0.426	<0.001 ***
FecA:Maternal age^2	-0.019	-0.024	-0.013	<0.001 ***
FecA:Region [LA]	0.088	-0.254	0.414	0.627
FecA:Region [MG]	-0.508	-0.796	-0.218	<0.001 ***
FecA:Region [NG]	-0.441	-0.700	-0.194	0.0017 **
FecA:Region [SG]	-0.241	-0.544	0.026	0.0901 .
FecA:Region [SI]	-0.181	-0.447	0.065	0.175
FecA:PopDens	-0.005	-0.009	0.000	0.0476 *

Bivariate model (c) of birth weight and female annual survival

	post.mean	l-95% CI	u-95% CI	pMCMC
CaptWt:Intercept	4.584	3.801	5.353	<0.001 ***
SurvA:Intercept	5.601	1.348	10.761	<0.001 ***
CaptWt:Calf sex [male]	-0.327	-0.398	-0.256	<0.001 ***
CaptWt:AgeHrs	0.016	0.015	0.017	<0.001 ***
CaptWt:Maternal age	0.448	0.377	0.524	<0.001 ***
CaptWt:Maternal age^2	-0.024	-0.028	-0.021	<0.001 ***
CaptWt:Repro [Naïve]	0.015	-0.151	0.166	0.855
CaptWt:Repro [SummerYeld]	0.490	0.355	0.611	<0.001 ***
CaptWt:Repro [TrueYeld]	0.411	0.303	0.510	<0.001 ***
CaptWt:Repro [Winter Yeld]	-0.201	-0.353	-0.063	0.005 **
CaptWt:Region [LA]	0.149	-0.098	0.425	0.284
CaptWt:Region [MG]	0.620	0.412	0.849	<0.001 ***
CaptWt:Region [NG]	0.140	-0.059	0.348	0.17
CaptWt:Region [SG]	0.842	0.602	1.054	<0.001 ***
CaptWt:Region [SI]	0.044	-0.172	0.240	0.667
CaptWt:PopDens	-0.002	-0.005	0.001	0.245
SurvA:Maternal age	0.137	-0.130	0.375	0.267
SurvA:Maternal age^2	-0.033	-0.054	-0.016	<0.001 ***
SurvA:PopDens	-0.005	-0.016	0.005	0.304

Bivariate model (d) of birth weight and female relative lifetime breeding success

	post.mean	l-95% CI	u-95% CI	eff.samp	pMCMC
CaptWt:Intercept	4.493	3.584	5.459	1177	<9e-04 ***
LBS:Intercept	1.035	0.864	1.214	1177	<9e-04 ***
CaptWt:Calf sex [male]	-0.331	-0.415	-0.247	1177	<9e-04 ***
CaptWt:AgeHrs	0.016	0.015	0.018	1177	<9e-04 ***
CaptWt:Maternal age	0.396	0.308	0.486	1249.4	<9e-04 ***
CaptWt:Maternal age^2	-0.022	-0.026	-0.018	1177	<9e-04 ***
CaptWt:Repro [Naïve]	-0.039	-0.223	0.144	1177	0.6882
CaptWt:Repro [SummerYeld]	0.462	0.328	0.613	1177	<9e-04 ***
CaptWt:Repro [TrueYeld]	0.449	0.334	0.577	1177	<9e-04 ***
CaptWt:Repro [Winter Yeld]	-0.210	-0.365	-0.048	971.7	0.0119 *
CaptWt:Region [LA]	0.064	-0.272	0.379	1177	0.6933
CaptWt:Region [MG]	0.593	0.341	0.865	1177	<9e-04 ***
CaptWt:Region [NG]	0.166	-0.070	0.399	1177	0.1614
CaptWt:Region [SG]	0.889	0.593	1.223	1177	<9e-04 ***
CaptWt:Region [SI]	-0.054	-0.312	0.173	1177	0.6525
CaptWt:PopDens	0.000	-0.005	0.004	1177	0.8343
